# Value of dual energy CT in post resuscitation coma. Differentiating contrast retention and ischemic brain parenchyma

**DOI:** 10.1016/j.radcr.2022.07.046

**Published:** 2022-08-04

**Authors:** Asra Nayab, Eelco F. Wijdicks, Patrick H. Luetmer, Vance T. Lehman

**Affiliations:** a Division of Neuroradiology Department of Radiology, Mayo Clinic Rochester, MN, USA; b Department of Neurology and Neurocritical Care, Mayo Clinic Rochester, MN, USA; c Radiology Education Office MA 2-00C Mayo Clinic 200 First Street SW Rochester, MN 55905, USA; d Division of Neurology and Neurocritical Care Mayo Clinic Rochester, MN, USA

**Keywords:** Dual-energy computed tomography (DECT), Virtual non-contrast (VNC), Brain ischemia, Contrast retention

## Abstract

Applications of dual-energy computed tomography and virtual non-contrast technique in neuroimaging are still emerging. While the role of DECT in differentiating parenchymal hemorrhage and contrast media after mechanical revascularization is well recognized, the value of DECT in evaluation of brain ischemia in post resuscitation patients who have received intravenous (IV) iodinated contrast is not well documented. We present a challenging case where DECT helped explain hyperattenuation in cortical grey matter and deep grey nuclei as well as cerebellar hemispheres in a comatose patient post cardiac arrest following massive pulmonary embolism.

## Introduction

Applications of dual-energy computed tomography (DECT) and virtual non-contrast (VNC) techniques in neuroimaging are still emerging. While the role of DECT in differentiating between brain parenchymal hemorrhage and leaked contrast media after mechanical revascularization is well recognized [1], the value of DECT in evaluation of brain ischemia in post resuscitation patients who have received intravenous (IV) iodine contrast is not well documented. We present a challenging case where DECT helped explain hyperattenuation in cortical grey matter and deep grey nuclei as well as cerebellar hemispheres in a comatose patient post cardiac arrest following massive pulmonary embolism.

## Case report

19-year-old female with no significant past medical history with only reported medication intake of oral contraceptive pills initially reported increasing dyspnea for 1-2 weeks preceding collapse with initial return of consciousness following bystander cardiopulmonary resuscitation. She subsequently lost consciousness, and Emergency Medical Services found her to be in ventricular fibrillation followed by pulseless electrical activity. She underwent pan-CT scanning with IV contrast earlier in the management which demonstrated massive pulmonary embolism resulting in right heart strain and manifestations of multi-organ failure. Within 24-hours of presentation the patient underwent percutaneous pulmonary artery thrombectomy and inferior vena cava filter placement. Serial head CTs performed are discussed below. Patient expired, following a short course, from multisystem failure.

## Brain imaging

**Fig. 1:**
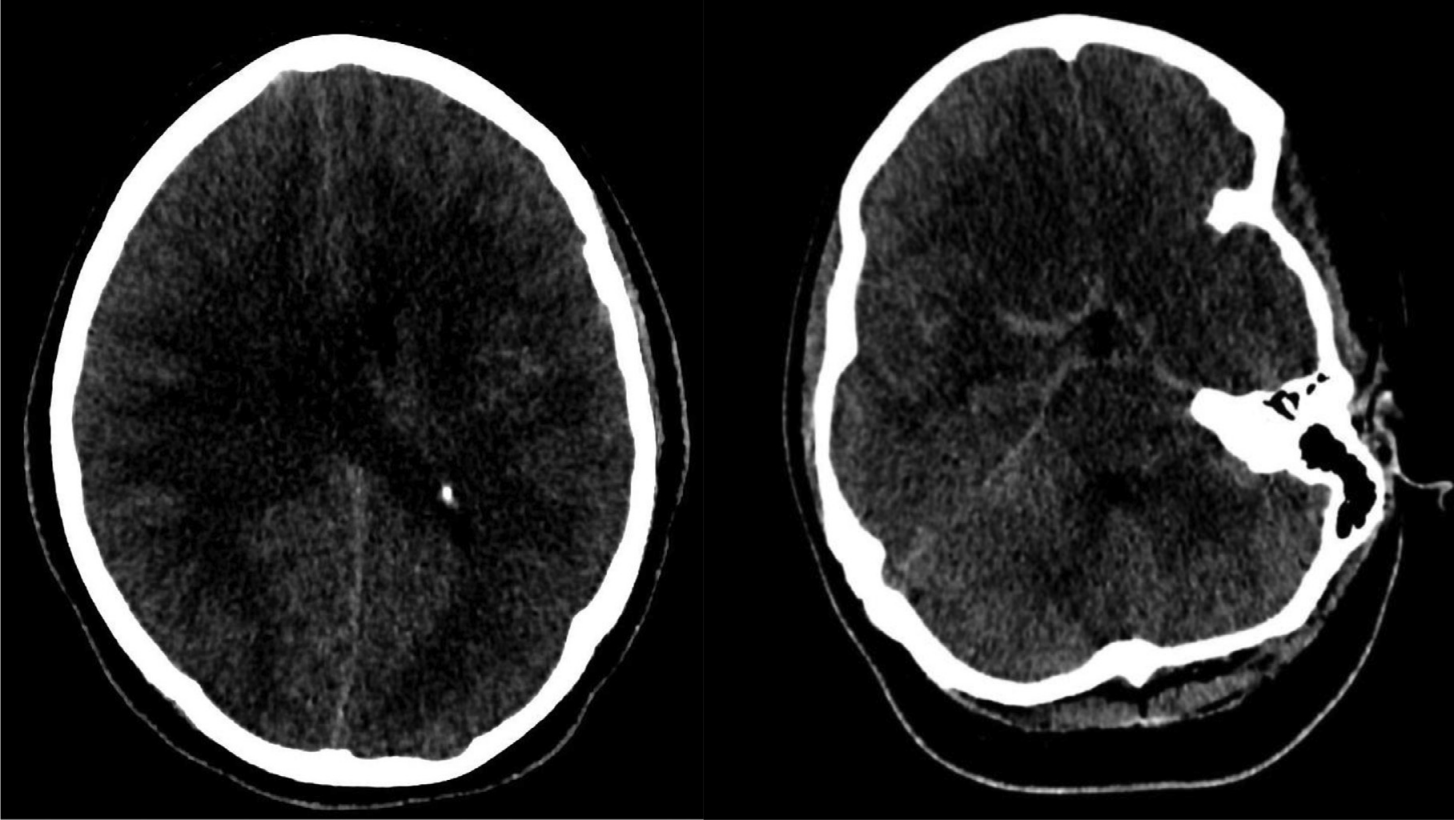
Axial non-contrast CT head images demonstrating areas of subarachnoid hyperdensities termed as pseudosubarachnoid appearance due to vascular engorgement secondary to diffuse cerebral edema and underlying history of rescusitation from cardiopulmonary arrest.

**Fig. 2:**
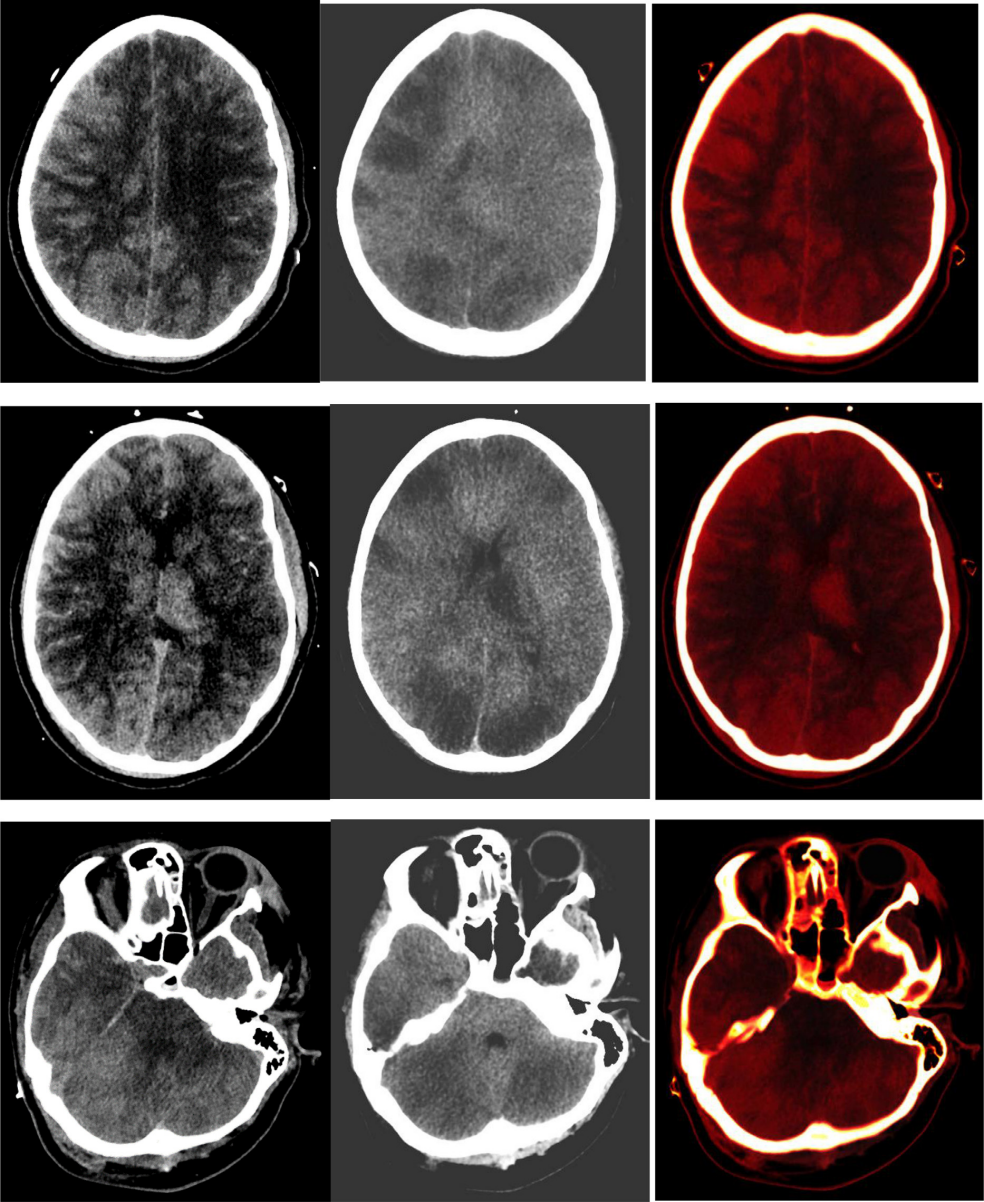
Left column: Non-contrast CT head demonstrates pronounced sulcal hyperdensity as well as hyperdense left thalamus, cortical and sub-cortical areas and cerebellum. Mean attenuation value was 103.17 HU in the right parasagittal parietal cortex and subcortical region, 109.25 Hounsfield unit (HU) in the left thalamus, and 112.27 HU in the right cerebellar hemisphere. Middle and right columns (Dual-Energy CT images): The right sided fused images of the brain demonstrate bright areas of contrast retention within the subarachnoid spaces as well as within patchy cortical and sub-cortical areas, left thalamus, and cerebellum. The middle subtracted virtual non-contrast (VNC) images demonstrate extensive low attenuation in areas of contrast retention consistent with widespread areas of infarction in a pattern consistent with diffuse hypoxic-ischemic injury.

Serial CT imaging of the head was obtained (Fig. 1) that demonstrated worsening diffuse cerebral edema and pseudo subarachnoid hemorrhage appearance of the cerebral sulci. A non-contrast CT head obtained less than 12 hours after initial post-contrast imaging demonstrated, in addition to more pronounced diffuse sulcal hyperdensity, indeterminate widespread patchy hyperattenuation of cortical and subcortical regions, left thalamus and cerebellum. These areas of patchy hyperdensities became increasingly hyperattenuating on subsequent non-contrast CT head imaging (Fig. 2). Given the reduced renal function and administration of 180 ml iohexol 350 mg iodine/ml intravenous contrast for pulmonary artery thrombectomy and inferior vena cava filter placement 17 hours prior and 130 ml iohexol 350 mg iodine/ml for CT angiogram of the head 10 hours prior to repeat CT head imaging, DECT with VNC images were obtained to differentiate hemorrhage from contrast retention. The fusion images demonstrated bright areas superimposed on the subarachnoid spaces as well as over the widespread areas of cortex and deep grey nuclei including the left thalamus, and cerebellum. These areas appeared hypodense on the subtracted images confirming iodine retention in areas of ischemic brain parenchyma.

## Discussion

DECT facilitates material differentiation based on different peak voltage acquisitions (high and low). Materials demonstrating equal Hounsfield densities at 120 kVp imaging can be distinguished by assessing energy dependent changes of attenuation of respective materials [2]. Introduced in 1973 [3], furthered in 1976 [4] and implemented in 2000s [5], [6], [7], various neurological and non-neurological applications of this technique have been described [8]. Wiggins et al. proved DECT utilization to differentiate small intracranial foci of hemorrhage from calcium [9]. DECT application in highlighting brain parenchyma at risk of hemorrhage following intra-arterial recanalization in stroke patients has been described [2,[10], [11], [12]]. Application of DECT for assessing ischemic tissue is on the horizon with myocardial ischemia and acute bowel ischemia currently studied non-neurological indications [13], [14], [15], [16]. To the best of our knowledge no literature has been published describing the value of DECT in assessing ischemic brain parenchyma in post-resuscitation comatose patients. We present such a case of a 19-year-old comatose patient from cardiac arrest and multi-organ failure following massive pulmonary embolism.

In our case, serial non-contrast CTs of the head demonstrated progressively increasing attenuation of the subarachnoid spaces that was attributed to pseudo subarachnoid hemorrhage appearance (vascular engorgement) secondary to severe diffuse cerebral edema and recent resuscitation from cardiopulmonary arrest, as has been previously described [17]. The presence of patchy extensive non-hemorrhage-like hyperattenuation of predominantly cortical and some subcortical areas, left thalamus and cerebellum on the latest non-contrast CT head, in the setting of previous IV iodinated contrast administration, was difficult to explain. Given the underlying presence of renal failure and recent IV contrast administration, subsequent CT was obtained using DECT with VNC technique to definitively assess the subarachnoid and parenchymal hyperattenuation. Brain parenchymal hyperattenuation following procedures utilizing IV contrast is a common finding that can be due to hemorrhage and/or contrast staining [18]. Endothelial injury results in blood-brain barrier disruption that allows contrast pooling into the interstitial compartment appearing as parenchymal hyperdensity on subsequent CT head exams [19]. Grey matter is vulnerable to ischemic insult due to higher metabolic demand. The injury evolves over the course of days to weeks with a usual underlying history of resuscitation following cardiac arrest in this age group [20,21]. Our case is unique in that the hyperattenuating parenchymal areas were extensive and not territorial as might be expected following revascularization, and lacked the typical intraparenchymal hemorrhagic appearance [22].

The VNC technique proved 2 things. First, the subarachnoid hyperattenuation was from contrast retention within the vasculature, and second and more importantly, the extensive hyperattenuating brain parenchymal areas that were bright on iodine maps were hypodense on the subtracted VNC images representing ischemic tissue retaining contrast. The VNC images confirmed underlying areas of brain infarction with extensive hypoattenuation in a pattern typical of diffuse hypoxic/ischemic insult. Hence DECT helped reveal widespread cerebral and cerebellar infarctions by confirming contrast retention in the areas of infarction.

## Conclusion

In conclusion, DECT with VNC is valuable in assessing brain parenchymal hyperdensities seen on a non-contrast CT in the setting of post resuscitation hypoxic/ischemic injury with prior history of IV iodine contrast administration. It differentiates contrast retention from hemorrhage and reveals underlying parenchymal infarction.

## Patient Consent

Written informed consent for all pertinent details of this case was obtained from patient's representative.

## References

[1] Tijssen M.P.M., Hofman P.A.M., Stadler A.A.R., van Zwam W., Graaf R.de, van Oostenbrugge R.J. (2014). The role of dual energy CT in differentiating between brain haemorrhage and contrast medium after mechanical revascularisation in acute ischaemic stroke. Eur Radiol.

[2] Postma A.A., Das M., Stadler A.A.R., Wildberger J.E. (2015). Dual-energy CT: what the neuroradiologist should know. Curr Radiol Rep.

[3] McCollough C.H., Leng S., Yu L., Fletcher J. (2015). Dual- and multi-energy CT: principles, technical approaches, and clinical applications. Radiology.

[4] Alvarez R.E., Macovski A. (1976). Energy-selective reconstructions in x-ray computerised tomography. Phys Med Biol.

[5] Kalender W., Bautz W., Felsenberg D., Sus C., Klotz E. (1987). Material-selective imaging and density measurement using the dual-energy method. I. Principles and methodology. Digitale Bilddiagnostik.

[6] Brooks R.A. (1977). A quantitative theory of the Hounsfield unit and its application to dual energy scanning. J Comput Assist Tomogr.

[7] Flohr T.G., McCollough C.H., Bruder H., Petersilka M., Gruber K., Sub C. (2006). First performance evaluation of a dual-source CT (DSCT) system. Eur Radiol.

[8] Patino M., Prochowski A., Agrawal M.D., Simeone F.J., Gupta R., Hahn P.F. (2016). Material separation using dual-energy CT: current and emerging applications. RadioGraphics.

[9] Wiggins W.F., Potter C.A., Sodickson A.D. (2020). Dual-energy CT to differentiate small foci of intracranial hemorrhage from calcium. Radiology.

[10] Almqvist H., Almqvist N.S., Holmin S., Mazya M.V. (2020). Dual-energy CT follow-up after stroke thrombolysis alters assessment of hemorrhagic complications. Front Neurol.

[11] Phan C.M., Yoo A.J., Hirsch J.A., Nogueira R.G., Gupta R. (2012). Differentiation of hemorrhage from iodinated contrast in different intracranial compartments using dual-energy head CT. Am J Neuroradiol.

[12] Morhard D., Ertl L., Gerdsmeier-Petz W., Ertl-Wagner B., Schulte-Altedorneburg G. (2014). Dual-energy CT immediately after endovascular stroke intervention: prognostic implications. Cardiovasc Interven Radiol.

[13] Lourenco P.D.M., Rawski R., Mohammed M.F., Khosa F., Nicolaou S., McLaughlin P. (2018). Dual-energy CT iodine mapping and 40-keV monoenergetic applications in the diagnosis of acute bowel ischemia. Am J Roentgenol.

[14] Obmann M.M., Punjabi G., Obmann V.C., Boll D.T., Heye T., Benz M.R. (2022). Dual-energy CT of acute bowel ischemia. Abdom Radiol.

[15] Ruzsics B., Lee H., Zwerner P.L., Gebregziabher M., Costello P., Schoepf U.J. (2008). Dual-energy CT of the heart for diagnosing coronary artery stenosis and myocardial ischemia-initial experience. Eur Radiol.

[16] Kang D.K., Schoepf U.J., Bastarrika G., Nance J.W., Abro J.A., Ruzsics B. (2010). Seminars in ultrasound, CT and MRI.

[17] Yuzawa H., Higano S., Mugikura S., Umetsu A., Murata T., Nakagawa A. (2008). Pseudo-subarachnoid hemorrhage found in patients with postresuscitation encephalopathy: characteristics of CT findings and clinical importance. Am J Neuroradiol.

[18] Byrne D., Walsh J.P., Schmiedeskamp H., Settecase F., Heran M.K.S., Niu B. (2020). Prediction of hemorrhage after successful recanalization in patients with acute ischemic stroke: improved risk stratification using dual-energy CT parenchymal iodine concentration ratio relative to the superior sagittal sinus. AJNR Am J Neuroradiol.

[19] Renú A., Amaro S., Laredo C., San R., Llull L., Lopez A. (2015). Relevance of blood–brain barrier disruption after endovascular treatment of ischemic stroke. Stroke.

[20] Vintila I., Roman-Filip C., Rociu C. (2010). Hypoxic-ischemic encephalopathy in adult. Acta Medica Transilvanica.

[21] Huang B.Y., Castillo M. (2008). Hypoxic-ischemic brain injury: imaging findings from birth to adulthood. Radiographics.

[22] Heit J.J., Iv M., Wintermark M. (2017). Imaging of intracranial hemorrhage. J Stroke.

